# Analysis of Healthcare Workers' Knowledge About Vital Sign Zero and Identify-Isolate-Inform (3I) System in the Diagnosis and Prevention of Infectious Diseases in Chinese Tertiary Hospitals

**DOI:** 10.3389/fpubh.2022.864197

**Published:** 2022-05-20

**Authors:** Zhijun Zhang, Yongjie Sha, Fengshi Jing, Weiming Tang, Shixing Tang

**Affiliations:** ^1^School of Public Health, Southern Medical University, Guangzhou, China; ^2^University of North Carolina at Chapel Hill Project-China, Guangzhou, China

**Keywords:** COVID-19, vital sign zero, 3I system, healthcare worker, infection disease control

## Abstract

**Objective:**

To explore the current knowledge and application of vital sign zero and the identify-isolate-inform (3I) system among healthcare workers in China in order to provide a reference for future improvement of healthcare workers' awareness of personal protection and prevention and control measures of infectious diseases.

**Methods:**

The questionnaire was used to investigate the basic information of health care workers, their knowledge and application of Vital sign zero and the 3I system. A total of 602 forms of health care workers from tertiary hospitals were randomly collected and included for analysis.

**Results:**

The survey showed that 45.30% and 57.30% of the healthcare workers from Chinese tertiary hospitals know about vital sign zero and 3I system while 51.80% and 57.30% of them applied these measures in their clinical practices. Logistics regression analysis results showed that healthcare workers aged 35 years old and below were less aware of vital sign zero than those above 50 years old (*OR* = *0.405, 95% CI: 0.174–0.942, P* = *0.036*). Compared with those in Northwest China, healthcare workers who worked in East China (*OR* = *0.147, 95% CI: 0.031–0.702, P* = *0.016)*, Central China (*OR* = *0.085, 95% CI: 0.018–0.403, P* = *0.002)*, Southwest China (*OR* = *0.083, 95% CI: 0.014–0.48, P* = *0.006)* and North China (*OR* = *0.201, 95% CI: 0.042–0.966, P* = *0.045*) were less aware of vital sign zero while the healthcare workers in Northeast China (*OR*=*9.714, 95% CI: 1.091–86.521, P* = *0.042)*, East China (*OR* = *18.049, 95% CI: 2.258–144.259, P* = *0.006)*, Central China (*OR* = *25.560, 95% CI: 3.210–203.502, P* = *0.002)*, South China (*OR* = *11.141, 95% CI: 1.395–88.947, P* = *0.023)*, Southwest China (*OR* = *23.200, 95% CI: 2.524–213.286, P* = *0.005)* and North China (*OR* = *14.078, 95% CI: 1.756–112.895, P* = *0.013*) had a better understanding of the 3I system than those in Northwest China. Healthcare workers with more than 20 years of working experience showed less knowledge of the 3I system than those with less than 5 years of working experience (*OR* = *0.409, 95% CI: 0.215–0.77, P* = *0.006*).

**Conclusion:**

The current levels of knowledge and application of vital sign zero and the 3I system in the healthcare workers of Chinese tertiary hospitals need to be improved. The concept of vital sign zero should be incorporated into the prevention triage system of infectious diseases.

## Background

The coronavirus disease 2019 (COVID-19) is an acute infectious disease caused by the novel severe acute respiratory syndrome coronavirus 2 (SARS-CoV-2), which is transmitted primarily by respiratory droplets and close contact. As of 24 April 2022, over 500 million confirmed cases and over six million deaths have been reported globally (1). Although COVID-19 vaccines are currently in use, prevention of SARS-CoV-2 infection is still most important as novel SARS-CoV-2 variants and breakthrough infections continue to occur.

As the main force in the fight against the epidemic, frontline healthcare workers in the hospital and emergency department play a critical role. They must strengthen their self-protection and enhance personal vigilance to avoid infection. They also paly important role in identifying and isolating patients who may have infectious diseases promptly. Triage usually requires healthcare workers to pay attention to the patient's vital signs, including respiration, pulse, blood pressure, and heart rate, etc. The outbreak and epidemic of severe acute respiratory syndrome (SARS) in 2003, Middle East respiratory syndrome (MERS) in South Korea in 2015, and the COVID-19, indicated that lack of knowledge about infectious diseases among healthcare workers and the failure to identify and isolate patients suffering from infectious diseases in the first instance may be associated with the expansion of infectious diseases. Therefore, Dr. Koening proposed to include the presence of infectious diseases as one of the vital signs in triage, called “Vital Sign Zero” (2). By incorporating the concept of Vital Sign Zero into the disease triage system, healthcare workers can maintain a high level of vigilance for infectious diseases, identify patients with infectious diseases on time, and reduce the transmission risk of infectious disease.

“Vital Sign Zero” means that at the beginning of triage, health care workers should first assess whether the patients are infected with infectious diseases or not and provide appropriate levels of personal protective equipment (PPE) to themselves. When a patient is found to have an infectious disease, he or she should be promptly isolated following the requirements of preventive and control measures (2, 3).

In addition, infectious disease control tools can help healthcare workers to better manage patients with infectious diseases. The Identify-Isolate-Inform system (3I Tool), originally conceived for the initial detection and management of Ebola virus infection and has been modified to prevent and control infectious diseases such as COVID-19, Zika, measles, mumps, and MERS (3–8). The 3I system helps healthcare workers identify patients with infectious diseases, master measures to isolate patients, and procedures for reporting infectious diseases promptly. It would be of great significance for the prevention and control of infectious diseases, including COVID-19 if healthcare workers could combine the 3I system with vital sign zero and implement them in their practice.

This study was conducted in tertiary hospitals in China with the aim of investigating the current knowledge and implementation about vital sign zero and 3I system among healthcare workers. The results will serve as a reference for improving the knowledge about personal protection and the ability of healthcare workers to respond to patients with infectious diseases.

## Methods

### Study Design, Procedure and Participants

The data used in this study was collected between March 3, 2021 and April 28, 2021. An online questionnaire was conducted through the Sojump (Sojump, Shanghai, China) website and included basic information (demographic characteristics of the participants, basic information of the medical institutions), behavioral characteristics, anxiety levels (the seven-item Generalized Anxiety Disorder Scale, GAD-7) (9), hospital triage application, healthcare workers' knowledge and application of vital sign zero and the 3I system. The questionnaires were distributed in collaboration with JMRE Academic, a medical related online platform. WeChat official accounts, WeChat group and Short messages were used to disseminate the survey link to health care workers on the platform. The inclusion criteria of the participants included: (1) healthcare workers, including doctors, nurses, medical technicians, pharmacists, logistical staff and administrative staff; (2) ≥18 years old; (3) voluntary participation in this study; (4) completing and submitting questionnaire forms and written informed consent. Eligible participants who completed the survey received a digital gift card of USD$7.

### Quality Control

The contents of the questionnaire (Table 1) were discussed and formulated by the full-time staff of the Institute For Global Health and STDs Center of Southern Medical University. The necessary options were set as mandatory answers to ensure the integrity of the questionnaire. The questionnaire is anonymous and does not involve personal privacy information, not including sensitive language. The questionnaire adopted the principle of voluntary filling and 100% of respondents completed the questionnaire. After the questionnaire was collected, the logical check was conducted to eliminate the questionnaire that did not meet the inclusion criteria. A total of 677 valid questionnaires were collected in this study, of which 602 were from the medical staff of tertiary hospitals, were included in the study, and 89% of the questionnaires finally met the inclusion criteria. All the 677 valid questionnaires were randomly collected from online survey and there was no significant difference in the major demographic characteristics between the 677 investigated subjects and the 602 selected objects *P* > *0.050* suggesting the 602 selected subjects were representative. The reliability analysis was conducted and showed that the Cronbach's alpha of the total questionnaire was 0.880 while the alpha values were 0.775 for vital sign zero dimension, 0.649 for 3I system dimension, and 0.780–0.884 between other dimensions, respectively. The validity analysis showed a KMO of 0.730 (*KMO* > *0.700*, Bartlett's Test: *P* < 0.001). These results indicated that the reliability and validity of the recalled questionnaire were satisfied.

**Table 1:** Variables and their assignments.

**Variables**	**The assignment**
**Demographic characteristics**	
Gender	0 = male, 1 = female
Age	0 = 50 years and above, 1 = 35-50 years, 2 = 35 years and under
Career years	0 = less than 5 years, 1 = 5-20 years, 2 = more than 20 years
Education	0 = Diploma, 1 = Undergraduate, 2 = Master, 3 = Doctor, 4 = Other
Occupation	0 = Doctor, 1 = Nurse, 2 = Medical technician, 3 = Pharmacist, 4 = Logistical staff, 5 = Administrative staff, 6 = Other
Professional title	0 = Senior, 1 = Middle, 2 = Elementary, 3 = Resident, 4 = Intern, 5 = Other
Have experience of fighting against infectious diseases	0 = no, 1 = yes
**Basic information of the medical institution**	
Regional distribution	0 = Northwest China, 1 = Northeast China, 2 = East China, 3 = Central China, 4 = South China, 5 = Southwest China, 6 = North China
**Cognitive situation**	
Have you ever heard of vital sign zero	0 = no, 1 = yes
Have you ever heard of 3I system	0 = no, 1 = yes
**The application situation**	
Have you ever applied vital sign zero in your work	0 = no, 1 = yes
Have you ever used 3I system in your work	0 = no, 1 = yes

### Statistical Analysis

The main research factors and variable assignment were shown in Table 1. All statistical analyses were performed using SPSS statistical software, version 26. The Chi-square test was used to identify potential predictive and associated factors. The logistic regression model was used to analyze the factors to affect healthcare workers' knowledge of vital sign zero and 3I system. The Wald two-sided 95% confidence interval (CI) was used. A two-sided *P* value <0.050 was considered statistically significant.

## Results

### Demographic Characteristics

In the study, 53.30% of the healthcare workers were female and 63.50% were below 35 years old. Furthermore, 44.90% had 5-20 years of work experience and 65.10% of them were physicians. In total 41.20% of the healthcare workers had experience in fighting against infectious diseases. Of note, 45.30% and 51.80% of the healthcare workers knew or had applied vital sign zero, respectively. In addition, 57.30% of the healthcare workers knew the 3I system, and 69.10% of them had applied the 3I system in their practices (Table 2).

**Table 2:** Demographic characteristics of participants from tertiary hospitals, China (*n*, %).

**Characteristics**		**Hospital location**								
	**Total**	**North China**	**Northeast**	**East China**	**Central China**	**South China**	**Southwest**	**Northwest**	***X^**2**^***	***P values***
			**China**				**China**	**China**		
	**(*n* = 602, %)**	**(*n* = 112, %)**	**(*n* = 24, %)**	**(*n* = 126, %)**	**(*n* = 163, %)**	**(*n* = 138, %)**	**(*n* = 27, %)**	**(*n* = 12, %)**		
**Gender**									*9.790*	*0.134*
Male	281 (46.70)	52 (46.40)	8 (33.30)	70 (55.60)	65 (39.90)	69 (50.00)	11 (40.70)	6 (50.00)		
Female	321 (53.30)	60 (53.60)	16 (66.70)	56 (44.40)	98 (60.10)	69 (50.00)	16 (59.30)	6 (50.00)		
**Age**									*11.730*	*0.477*
35 and below	382 (63.50)	72 (64.30)	17 (70.80)	84 (66.70)	109 (66.90)	76 (55.10)	15 (55.60)	9 (75.00)		
35-50	194 (32.20)	36 (32.10)	7 (29.20)	39 (31.00)	45 (27.60)	55 (39.90)	10 (37.00)	2 (16.70)		
50 and above	26 (4.30)	4 (3.60)	0	3 (2.40)	9 (5.50)	7 (5.10)	2 (7.40)	1 (8.30)		
**Career years**									*15.470*	*0.118*
Within 5 years	284 (47.20)	53 (47.30)	10 (41.70)	63 (50.00)	85 (52.10)	55 (39.90)	11 (40.70)	7 (58.30)		
5-20 years	270 (44.90)	51 (45.50)	13 (54.20)	57 (45.20)	69 (42.30)	62 (44.90)	13 (48.10)	5 (41.70)		
More than 20 years	48 (8.00)	8 (7.10)	1 (4.20)	6 (4.80)	9 (5.50)	21 (15.20)	3 (11.10)	0		
**Education**									*90.590*	* <0.001*
Diploma	13 (2.20)	0	0	2 (1.60)	6 (3.70)	5 (3.60)	0	0		
Undergraduate	208 (34.60)	27 (24.10)	5 (20.80)	35 (27.80)	50 (30.70)	82 (59.40)	5 (18.50)	4 (33.30)		
Master	228 (37.90)	50 (44.60)	16 (66.70)	58 (46.00)	47 (28.80)	35 (25.40)	17 (63.00)	5 (41.70)		
Doctor	152 (25.20)	35 (31.30)	3 (12.50)	31 (24.60)	60 (36.80)	15 (10.90)	5 (18.50)	3 (25.00)		
Other	1 (0.10)	0	0	0	0	1 (0.70)	0	0		
**Occupation**									*131.590*	* <0.001*
Doctor	392 (65.10)	82 (73.20)	18 (75.00)	89 (70.60)	97 (59.50)	84 (60.90)	17 (63.00)	5 (41.70)		
Nurse	78 (13.00)	8 (7.10)	2 (8.30)	9 (7.10)	46 (28.20)	9 (6.50)	2 (7.40)	2 (16.70)		
Medical technician	32 (5.30)	9 (8.00)	2 (8.30)	8 (6.30)	9 (5.50)	1 (0.70)	2 (7.40)	1 (8.30)		
Pharmacist	21 (3.50)	3 (2.70)	1 (4.20)	10 (7.90)	2 (1.20)	4 (2.90)	0	1 (8.30)		
Logistical staff	7 (1.20)	0	0	1 (0.80)	1 (0.60)	4 (2.90)	0	1 (8.30)		
Administrative staff	26 (4.30)	6 (5.40)	1 (4.20)	5 (4.00)	7 (4.30)	3 (2.20)	2 (7.40)	2 (16.70)		
Other	46 (7.60)	4 (3.60)	0	4 (3.20)	1 (0.60)	33 (23.90)	4 (14.80)	0		
**Title**									*154.990*	* <0.001*
Senior	119 (19.80)	25 (22.30)	5 (20.80)	32 (25.40)	38 (23.30)	12 (8.70)	5 (18.50)	2 (16.70)		
Middle	201 (33.40)	41 (36.60)	6 (25.00)	40 (31.70)	76 (46.60)	22 (15.90)	10 (37.00)	6 (50.00)		
Elementary	129 (21.40)	24 (21.40)	5 (20.80)	27 (21.40)	39 (23.90)	24 (17.40)	7 (25.90)	3 (25.00)		
Resident	58 (9.60)	14 (12.50)	7 (29.20)	16 (12.70)	6 (3.70)	14 (10.10)	1 (3.70)	0		
Intern	76 (12.60)	6 (5.40)	1 (4.20)	8 (6.30)	3 (1.80)	54 (39.10)	3 (11.10)	1 (8.30)		
Other	19 (3.20)	2 (1.80)	0	3 (2.40)	1 (0.60)	12 (8.70)	1 (3.70)	0		
Experience in fighting against infectious diseases	248 (41.20)	43 (38.40)	5 (20.80)	44 (34.90)	117 (71.80)	28 (20.30)	5 (18.50)	6 (50.00)	*101.950*	* <0.001*
Know the concept of vital sign zero	273 (45.30)	57 (50.90)	7 (29.20)	74 (58.70)	50 (30.70)	61 (44.20)	14 (51.90)	10 (83.30)	*34.710*	* <0.001*
Application of vital sign zero	312 (51.80)	62 (55.40)	10 (41.70)	72 (57.10)	77 (47.20)	64 (46.40)	18 (66.70)	9 (75.00)	*10.960*	*0.090*
Know the concept of the 3I system	345 (57.30)	61 (54.50)	16 (66.70)	62 (49.20)	112 (68.70)	81 (58.70)	12 (44.40)	1 (8.30)	*26.970*	* <0.001*
Application of the 3I system	416 (69.10)	82 (73.20)	17 (70.80)	93 (73.80)	105 (64.40)	86 (62.30)	22 (81.50)	11 (91.70)	*11.340*	*0.070*

### Regional Distribution of the Tertiary Hospitals in This Study

The regions of the hospitals where the healthcare workers worked were divided by the seven administrative geographic divisions in China, in which 163 (27.00%) healthcare workers were from Central China, 138 (23.00%) from South China, 126 (21.00%) from East China, 112 (19.00%) from North China, and 27 (4.50%), 24 (4.00%), and 12 (2.00%) from Southwest, Northeast, and Northwest China, respectively (Table 2). Among the healthcare workers from different regions, the significant differences were observed with respect to education (*X*^2^ = *90.590, P*<*0.001*), occupation (*X*^2^ = *131.590, P*<*0.00*1), title (*X*^2^ = *154.990, P*<*0.001*), experience in fighting against infectious diseases (*X*^2^ = *101.950, P*<*0.001*), knowledge of vital sign zero (*X*^2^ = *34.710, P*<*0.001*), and knowledge of the 3I system (*X*^2^ = *26.970, P*<*0.001*, Table 2).

### Factors Associated With the Levels of Knowledge of Vital Sign Zero

Significant difference was found between the healthcare workers who knew or did not know the concept of vital sign zero with respect to gender (*X*^2^ = *4.260, P* = *0.039*), age (*X*^2^ = *6.290, P* = *0.043*), duration of career (*X*^2^ = *6.240, P* = *0.044*), hospital location (*X*^2^ = *34.710, P*<*0.001*), the experience of fighting against infectious diseases (*X*^2^ = *4.300, P* = *0.038*) and the application of vital sign zero (*X*^2^ = *182.770, P*<*0.001*, Table 3).

**Table 3:** Knowledge about vital sign zero among health care workers during COVID-19 pandemic.

**Characteristics**	**Vital sign zero awareness (** ***n*** **, %)**		***X^**2**^***	***P values***
	**Aware (*n* = 273, %)**	**Unaware (*n* = 329, %)**		
**Gender**			*4.260*	*0.039*
Male	140 (51.30)	141 (42.90)		
Female	133 (48.70)	188 (57.10)		
**Age**			*6.290*	*0.043*
35 and below	160 (58.60)	222 (67.50)		
35-50	97 (35.50)	97 (29.50)		
50 and above	16 (5.90)	10 (3.00)		
**Career years**			*6.240*	*0.044*
Within 5 years	118 (43.20)	166 (50.50)		
5-20 years	126 (46.20)	144 (43.80)		
More than 20 years	29 (10.60)	19 (5.80)		
**Education**			*5.590*	*0.227*
Diploma	5 (1.80)	8 (2.40)		
Undergraduate	100 (36.60)	108 (32.80)		
Master	110 (40.30)	118 (35.90)		
Doctor	58 (21.20)	94 (28.60)		
Other	0	1 (0.30)		
**Occupation**			*10.390*	*0.103*
Doctor	173 (63.40)	219 (66.60)		
Nurse	31 (11.40)	47 (14.30)		
Medical technician	11 (4.00)	21 (6.40)		
Pharmacist	13 (4.80)	8 (2.40)		
Logistical staff	5 (1.80)	2 (0.60)		
Administrative staff	13 (4.80)	13 (4.00)		
Other	27 (9.90)	19 (5.80)		
**Title**			*7.150*	*0.210*
Senior	46 (16.80)	73 (22.20)		
Middle	100 (36.60)	101 (30.70)		
Elementary	54 (19.80)	75 (22.80)		
Resident	23 (8.40)	35 (10.60)		
Intern	40 (14.70)	36 (10.90)		
Other	10 (3.70)	9 (2.70)		
**Hospital location**			*34.710*	* <0.001*
North China	57 (20.90)	55 (16.70)		
Northeast China	7 (2.60)	17 (5.20)		
East China	74 (27.10)	52 (15.80)		
Central China	50 (18.30)	113 (34.30)		
South China	61 (22.30)	77 (23.40)		
Southwest China	14 (5.10)	13 (4.00)		
Northwest China	10 (3.70)	2 (0.60)		
Experience in fighting against infectious diseases	100 (36.60)	148 (45.00)	*4.300*	*0.038*
Application of vital sign zero	224 (82.10)	88 (26.70)	*182.770*	* <0.001*

Logistic regression analysis with stepwise regression forward selection (based on likelihood ratio test) method showed that age and hospital location were the main factors associated with the knowledge of vital sign zero of healthcare workers (**Table 5**). Among them, people aged 35 years old and below were less aware of vital sign zero than those above 50 years old. Compared with those in Northwest China, healthcare workers who worked in East China, Central China, Southwest China and North China were less aware of vital sign zero. The corresponding odds ratios (OR) were *0.147 (95% CI: 0.031–0.702, P* = *0.016), 0.085 (95% CI: 0.018–0.403, P* = *0.002), 0.083 (95% CI: 0.014–0.48, P* = *0.006)* and *0.201 (95% CI: 0.042–0.966, P* = *0.045*), respectively.

### Factors Associated With the Knowledge of 3I System

Significant difference was found between the healthcare workers who knew or did not know the concept of the 3I system and healthcare workers in gender (*X*^2^ = *6.170, P* = *0.013*), career years (*X*^2^ = *6.940, P* = *0.031*), occupation (*X*^2^ = *12.770, P* = *0.044*), hospital location (*X*^2^ = *26.970, P*<*0.001*), and the application of 3I system (*X*^2^ = *80.800, P*<*0.001*, Table 4).

**Table 4:** Knowledge about 3I system among health care workers during COVID-19 pandemic.

**Characteristics**	**3I system awareness (** ***n*** **, %)**		***X^**2**^***	***P values***
	**Aware (*n* = 345, %)**	**Unaware (*n* = 257, %)**		
**Gender**			*6.170*	*0.013*
Male	146 (42.30)	135 (52.50)		
Female	199 (57.70)	122 (47.50)		
**Age**			*3.970*	*0.138*
35 and below	223 (64.60)	159 (61.90)		
35–50	112 (32.50)	82 (31.90)		
50 and above	10 (2.90)	16 (6.20)		
Career years			*6.940*	*0.031*
Within 5 years	170 (49.30)	114 (44.40)		
5–20 years	156 (45.20)	114 (44.40)		
More than 20 years	19 (5.50)	29 (11.30)		
**Education**			*3.090*	*0.523*
Diploma	9 (2.60)	4 (1.60)		
Undergraduate	125 (36.20)	83 (32.30)		
Master	123 (35.70)	105 (40.90)		
Doctor	87 (25.20)	65 (25.30)		
Other	1 (0.30)	0		
**Occupation**			*12.770*	*0.044*
Doctor	222 (64.30)	170 (66.10)		
Nurse	54 (15.70)	24 (9.30)		
Medical technician	21 (6.10)	11 (4.30)		
Pharmacist	8 (2.30)	13 (5.10)		
Logistical staff	2 (0.60)	5 (1.90)		
Administrative staff	16 (4.60)	10 (3.90)		
Other	22 (6.40)	24 (9.30)		
**Title**			*4.010*	*0.548*
Senior	70 (20.30)	49 (19.10)		
Middle	109 (31.60)	92 (35.80)		
Elementary	74 (21.40)	55 (21.40)		
Resident	39 (11.30)	19 (7.40)		
Intern	44 (12.80)	32 (12.50)		
Other	9 (2.60)	10 (3.90)		
**Hospital location**			*26.970*	* <0.001*
North China	61 (17.70)	51 (19.80)		
Northeast China	16 (4.60)	8 (3.10)		
East China	62 (18.00)	64 (24.90)		
Central China	112 (32.50)	51 (19.80)		
South China	81 (23.50)	57 (22.20)		
Southwest China	12 (3.50)	15 (5.80)		
Northwest China	1 (0.30)	11 (4.30)		
Experience in fighting against infectious diseases	144 (41.70)	104 (40.50)	*0.100*	*0.754*
Application of the 3I system	188 (54.50)	228 (88.70)	*80.800*	* <0.001*

Logistic regression analysis showed that career years and hospital location were the main factors affecting the knowledge of the 3I system (Table 5). Compared with those who worked in Northwest China, healthcare workers who worked in Northeast China, East China, Central China, South China, Southwest China and North China were had a better understanding of the 3I system with the corresponding OR of *9.714 (95% CI: 1.091–86.521, P* = *0.042), 18.049 (95% CI: 2.258–144.259, P* = *0.006), 25.560 (95% CI: 3.210–203.502, P* = *0.002), 11.141 (95% CI: 1.395–88.947, P* = *0.023), 23.200 (95% CI: 2.524–213.286, P* = *0.005)* and *14.078 (95% CI: 1.756–112.895, P* = *0.013*), respectively. Healthcare workers with more than 20 years of working experience had less knowledge of the 3I system than those with less than 5 years of working experience, and the OR was *0.409 (95% CI: 0.215–0.77, P* = *0.006)*.

**Table 5:** Logistic regression models of factors influencing the cognition of Vital sign zero and 3I system among health care workers from tertiary hospitals, China, 2021, (*N* = 602).

**Variables**	**β**	**SE**	**Wald**	***P values***	***OR(95%CI)***
**Vital sign zero**					
**Age**					
50 and above	(ref)
35–50	−0.558	0.442	1.593	*0.207*	*0.573(0.241–1.361)*
35 and below	−0.904	0.431	4.402	*0.036*	*0.405(0.174–0.942)*
**Hospital location**					
Northwest China	(ref)
Northeast China	−1.616	0.87	3.453	*0.063*	*0.199(0.036–1.093)*
East China	−1.917	0.798	5.775	*0.016*	*0.147(0.031–0.702)*
Central China	−2.469	0.796	9.61	*0.002*	*0.085(0.018–0.403)*
South China	−1.27	0.799	2.528	*0.112*	*0.281(0.059–1.344)*
Southwest China	−2.492	0.899	7.683	*0.006*	*0.083(0.014–0.482)*
North China	−1.604	0.801	4.015	*0.045*	*0.201(0.042–0.966)*
**3I system**					
**Career years**					
Within 5 years	(ref)
5–20 years	−0.092	0.177	0.267	*0.605*	*0.912(0.644–1.292)*
More than 20 years	−0.894	0.327	7.462	*0.006*	*0.409(0.215–0.777)*
**Hospital location**					
Northwest China	(ref)
Northeast China	2.274	1.116	4.153	*0.042*	*9.714(1.091–86.521)*
East China	2.893	1.06	7.442	*0.006*	*18.049(2.258–144.259)*
Central China	3.241	1.059	9.375	*0.002*	*25.560(3.210–203.502)*
South China	2.411	1.06	5.173	*0.023*	*11.141(1.395–88.947)*
Southwest China	3.144	1.132	7.716	*0.005*	*23.200(2.524–213.286)*
North China	2.645	1.062	6.199	*0.013*	*14.078(1.756–112.895)*

## Discussion

In this study, we found that the awareness rate of healthcare workers in Chinese tertiary hospitals was 45.30% and 57.30% for vital sign zero and 3I system, respectively while 51.80% and 57.30% of them applied these measures in their clinical practice. These results indicated that the awareness rates of both vital sign zero and the 3I system were low. The prevention knowledge and the system for managing infectious diseases should be further improved.

We further identified that healthcare workers who worked in East China, Central China, Southwest China and North China had poorer knowledge of vital sign zero, suggesting that hospitals in these areas should strengthen training on vital sign zero related knowledge. Strengthening and improving training on the concept of vital sign zero can help improve healthcare workers' awareness of self-protection, avoid infecting infectious diseases during triage and consultation.

As COVID-19 is pandemic and now is still spreading worldwide, healthcare workers must be highly cautious to patients who may suffer from infectious diseases when treating patients. Therefore, to be familiar with the concept of vital sign zero and to apply them appropriately are very important. To know about whether patients suffer from infectious diseases should be the first step in triage and consultation in order to protect healthcare workers' own safety and to avoid nosocomial infection. In addition, logistics regression results showed that healthcare workers aged 35 years old and below have a lower cognition level of vital sign zero than those aged 50 years old and above probably because senior healthcare workers have higher academic titles, rich experience in infectious disease prevention and control, and pay more attention to personal protection.

The logistic regression results of healthcare workers' cognition of the 3I system showed that the cognition of the 3I system of healthcare workers who worked in Northeast China, East China, Central China, South China, Southwest China and North China had a better understanding of the 3I system, suggesting that the hospitals located in these areas are more experienced in the process of identifying, isolating, and reporting patients with infectious diseases, which may be related to the rich experience of fighting the epidemic. Hospitals should continue to strengthen the training and application of the 3I system to increase the application rate of the 3I system among healthcare workers. Healthcare workers with more than 20 years of working experience need to strengthen training on the 3I system. Law of the People's Republic of China on the Prevention and Treatment of Infectious Diseases (10) requires any individual who discovers a patient of an infectious disease or a suspected patient of an infectious disease shall promptly report to the nearby disease prevention and control institution or medical institution. 3I system, which was originally used to prevent and control the Ebola virus infection and outbreaks, is a tool for early detection and prevention of disease spread. The 3I system is flexible and has been modified to apply to many infectious diseases, such as COVID-19, Zika, measles, mumps, and MERS with high satisfiction (4–8). The concept and specific methods of the 3I system can be applied to assist healthcare workers in identifying, isolating, and reporting patients with infectious diseases in an orderly manner, arranging patients to the appropriate isolation areas, and reporting them to the relevant departments in a timely and accurate manner, providing relief to patients while avoiding the occurrence of nosocomial infection and preventing the further spread and prevalence of infectious diseases. At present, the 3I system is mainly used to make posters or small cards that can be posted to facilitate healthcare workers to quickly understand the diagnosis and differential diagnosis of new infectious diseases and to more easily detect potential infectious disease patients (3).

The results of our study showed that there were statistically significant differences in education, occupation, titles, the experience of fighting against infectious disease, vital sign zero, and 3I system awareness among healthcare workers who worked in different regions of China. These results suggested that when hospitals strengthen the training of healthcare workers on the knowledge of vital sign zero and the 3I system, they should develop refined training programs among healthcare workers with different occupations, titles, and experience in fighting epidemics and cognitive status, and conduct different levels of training among the health workers, and improve the quality of training about the knowledge and application of vital sign zero and 3I system.

In this study, we analyzed the awareness level of healthcare workers in China tertiary hospitals and the factors influencing their knowledge of vital sign zero and the 3I system and investigated the current situation of healthcare workers in tertiary hospitals. Our results can provide a reference for hospitals to improve the corresponding infectious disease control measures and related knowledge training. The healthcare workers of tertiary hospitals are relatively more educated than other hospitals and have better systems than other lower-level hospitals; therefore, the healthcare workers' knowledge of vital sign zero and the 3I system may be relatively rich in this study. At the same time, tertiary hospitals represent the high level of medical care facilities in China. However, the level of awareness of vital sign zero and 3I system among healthcare workers in tertiary hospitals still needs to be improved. It means that similar training and improvments are highly needed in the healthcare workers in other lower-level hospitals in China.

Our study has limitations. First, vital zero sign and 3I system are new concepts although the similar measures or system to prevent infectious diseases exist or have been implemented in the majority of tertiary hospitals in China. There is no universal training program for the whole country. In our study, we did not investigate if the regions have different curricula or graduate programs. Therefore, the reasons for the difference of hospitals from different regions remain to be elucidated. Second, there were no other similar studies conducted in China about vital sign zero and the identify-isolate-inform (3I) system. We could not conduct specific comparison about the awareness rates in China. Third, since this study was conducted during the COVID-19 epidemic, we could only obtain information on the awareness level of vital sign zero and the 3I system among healthcare workers during the epidemic, and we lacked the comparison of awareness levels before and after the epidemic.

## Conclusion

The current levels of knowledge and application of vital sign zero and the 3I system in the healthcare workers of Chinese tertiary hospitals need to be improved. The concept of vital sign zero should be incorporated into the prevention triage system of infectious diseases. Healthcare workers should know about and apply the 3I system to strengthen their awareness of personal protection to prevent the spread of infectious diseases and to avoid nosocomial infections.

## Data Availability Statement

The original contributions presented in the study are included in the article/Supplementary material, further inquiries can be directed to the corresponding author.

## Ethics Statement

Ethical review and approval was not required for the study on human participants in accordance with the local legislation and institutional requirements. The patients/participants provided their written informed consent to participate in this study.

## Author Contributions

ZZ, YS, FJ, WT, and ST contributed to conception and design of the study. ZZ organized the database and wrote the first draft of the manuscript. ZZ and YS performed the statistical analysis. ZZ YS, FJ, WT, and ST wrote sections of the manuscript. All authors contributed to manuscript revision, read, and approved the submitted version.

## Conflict of Interest

The authors declare that the research was conducted in the absence of any commercial or financial relationships that could be construed as a potential conflict of interest.

## Publisher's Note

All claims expressed in this article are solely those of the authors and do not necessarily represent those of their affiliated organizations, or those of the publisher, the editors and the reviewers. Any product that may be evaluated in this article, or claim that may be made by its manufacturer, is not guaranteed or endorsed by the publisher.
